# R&D Incentives for Neglected Diseases

**DOI:** 10.1371/journal.pone.0050835

**Published:** 2012-12-19

**Authors:** Nicola Dimitri

**Affiliations:** 1 Department of Political Economy and Statistics, University of Siena, Siena, Italy; 2 Maastricht School of Management, Maastricht, Holland; 3 IMT Lucca, Lucca, Italy; Universidad Veracruzana, Mexico

## Abstract

Neglected diseases are typically characterized as those for which adequate drug treatment is lacking, and the potential return on effort in research and development (R&D), to produce new therapies, is too small for companies to invest significant resources in the field. In recent years various incentives schemes to stimulate R&D by pharmaceutical firms have been considered. Broadly speaking, these can be classified either as ‘push’ or ‘pull’ programs. Hybrid options, that include push and pull incentives, have also become increasingly popular. Supporters and critics of these various incentive schemes have argued in favor of their relative merits and limitations, although the view that no mechanism is a perfect fit for all situations appears to be widely held. For this reason, the debate on the advantages and disadvantages of different approaches has been important for policy decisions, but is dispersed in a variety of sources. With this in mind, the aim of this paper is to contribute to the understanding of the economic determinants behind R&D investments for neglected diseases by comparing the relative strength of different incentive schemes within a simple economic model, based on the assumption of profit maximizing firms. The analysis suggests that co-funded push programs are generally more efficient than pure pull programs. However, by setting appropriate intermediate goals hybrid incentive schemes could further improve efficiency.

## Introduction

Neglected diseases are typically characterised as those for which adequate drug treatment is lacking, because the potential return on investment in research and development (R&D), needed to produce new treatments, is too small to induce companies to employ significant resources in the field. As a result a large number of patients lack appropriate therapies, though many of such diseases can cause death or very poor quality of life. Examples include infectious diseases that predominantly affect the developing world, such as African trypanosomiasis and schistosomiasis, as well as rare diseases affecting also people in the more developed countries.

Mobilizing public and private institutions, researchers, and pharmaceutical firms to engage in R&D for neglected diseases has been the focus of considerable effort and funding in recent years. In particular, multiple public–private partnerships (PPPs), such as the Special Programme for Research and Training in Tropical Diseases (TDR), the Medicines for Malaria Venture (MMV), Drugs for Neglected Diseases (DNDi), and the Global Alliance for TB Drug Development, have been founded to spur R&D and drug delivery effort for infectious diseases. There have also been major influxes of funding, for example, from the Bill and Melinda Gates Foundation, as well as from national governments [1],[2],[3],[4],[5],[6],[7].

Various incentive mechanisms have been associated with such initiatives [8],[9],[10],[11],[12],[13] starting with the 1983 US legislation on Orphan Drugs, later on also enacted by other countries to fight rare diseases. Broadly speaking, these can be classified either as ‘push’ or ‘pull’ programmes. Push programmes have a direct impact on R&D expenditures, supporting drug discovery, and often take the form of upfront research grants, from public institutions or charities to pharmaceutical firms. On the other hand, pull incentives stimulate research effort indirectly, by enhancing the revenue potential and/or lowering delivery costs. Examples include differential pricing, advanced market commitments (AMC) and prize mechanism proposals, an important route to implement the principle of “delinking” price from R&D costs, recently advocated by the Consultative Expert Working Group (CEWG) on R&D of the World Health Organization [14]. Hybrid options that include push and pull incentives have also become increasingly adopted in recent years, a notable example of which are Orphan Drugs legislations.

Supporters and critics of these various incentive schemes have argued in favour of their relative merits and limitations, although the view that no mechanism is a perfect fit for all situations appears to be widely held. For this reason, though the debate on the advantages and disadvantages of different approaches has been important for policy decisions, it is dispersed in a variety of sources and not always easy to summarise. With this in mind, the aim of this paper is to contribute to the understanding of the economic determinants behind R&D investments for neglected diseases through the presentation of economic models of various incentive schemes. More specifically, the main goal of such models is to provide a formal platform where comparison of different incentive programs for R&D investments could be made within a unified framework. Though most of the analysis will be based on the standard assumption of profit maximising pharmaceutical companies, in the supplementary material we shall also consider alternative goals such as productivity maximisation, as well as zero-profit R&D investment levels that may characterise non-commercial institutions developing new drugs. As with every model our goal is not to provide explanations for each single, and different, case but rather to gain insights on the main underlying forces behind alternative incentive schemes. Indeed, as we shall see, some of the results are not obvious. Though inspired by neglected diseases, the analysis is more general and the main findings can also apply to corporate R&D incentive schemes, concerning any kind of treatment and disease. Finally, while the main text shortly presents the fundamental insights of the work, a detailed analysis is contained in the supporting information S1.

## Results

### R&D investment with no incentives

To illustrate the economic basis for decisions by companies about levels of investment in R&D for neglected diseases, a simple numerical example can be used, starting with the case of a profit maximizing company with no external R&D incentives (see the supporting information S1 for a more general analysis).

Consider a pharmaceutical company which, for simplicity and no major loss of generality, can only undertake four levels of R&D investments *C* (in billions $), that appear on the top row of Table 1.

**Table 1:** The firm expected profit with no incentives.

	C = 0	C = 0.2	C = 0.4	C = 0.6	Comment
**Expected Profit** *	0	−0.05	−0.2	−0.34	No incentive to invest in R&D

* The expected profit term includes no funding by external sponsors. The net revenue *R* from a successful product is *0.3*. The probabilities of successful R&D investment for the firm (*C = 0*, *C = 0.2*, *C = 0.4* and *C = 0.6*) are, respectively, *0, 0.5, 0.66* and *0.85*.

Each such R&D level has a corresponding success probability *p(C)*, that reflects the assumption of decreasing returns on R&D investment. For instance, if R&D investment is doubled then success probability increases but less than twice the initial value. In the supporting information S1 other assumptions concerning returns from investments will also be considered.

Assuming that perspective profits (net revenues), that is revenues minus production and delivery costs, from a successful investment are low (for example *R = 0.3*), and no additional external funds, Table 1 shows the expected profit associated to the four R&D levels (for details see the supporting information S1). Though a very important issue with infectious and tropical diseases, it is not a goal of this paper to discuss how drug delivery can be achieved and at what costs. We simply assume that it could always be possible at some costs, and incorporated in *R*. Finally, perspective revenues may be low either because demand is potentially high but individuals are poor and cannot pay for treatment, as in the case of infectious diseases, or because the disease is rare and the number of affected individuals low.

In this case no investment, *C = 0* is the best plan for the company then, respectively, *C = 0.2* and *C = 0.4* with the highest *C = 0.6* being the least preferred. As a consequence, the company would not engage in R&D and the disease remains neglected. Notice that this would be so even if the goal is to invest in R&D as long as profits are non-negative.

### Comparing R&D incentive schemes

Suppose now that an external sponsor wishes to support R&D to find a treatment for the neglected disease, and has an available budget of *B = 1.2*. The sponsor may use the whole budget, or part of it, to stimulate the firm R&D through a funding function made of a fixed sum *F*, and a component *f(C)* related to *C∶F+f(C)* (see the supporting information S1 for more details).

If the aim of the funder is to maximize the probability of discovery which, given the shape of the success probability typically coincides with maximizing the total amount invested in R&D, the problem for the sponsor is to choose how the money should be transferred to the company. In what follows we model the outcome of various options using push incentives, pull incentives or push– pull hybrid strategies, with either variable or constant funding from the sponsor.

#### Variable funding

With variable funding, the main findings of the analysis are summarised by Table 2 (see the supporting information S1 for more details).

**Table 2:** Comparing push and pull mechanisms with variable funding *F(C) = C.*

	Expected profit*				Comment
Scheme	C = 0	C = 0.2	C = 0.4	C = 0.6	
**Push**	0	0.05	0.2	0.51	Optimal to choose the maximum R&D level
**Pull**	0	0.05	0.07	0.16	Optimal to choose the maximum R&D level

* The expected profit term includes the funding provided by the sponsor, whether or not it is actually spent on R&D by the firm. The net revenue *R* from a successful product is *0.3*. The probabilities of successful R&D investment for the firm (*C = 0*, *C = 0.2*, *C = 0.4* and *C = 0.6*) are, respectively, *0, 0.5, 0.66* and *0.85*.

In this model with profit maximisation and variable funding (in the example *F(C) = C*), the expected profit columns in Table 2 indicate that both push and pull incentives induce the highest possible level of R&D by the company, but that push schemes are more efficient as they provide a larger expected payoff to the firm.

A point is noteworthy here. To induce the highest level of R&D, the sponsor does not need to use the whole budget. Indeed, with push incentives, the maximum level of R&D could have been induced by spending less than C = 0.6 (for more on this see the supporting information S1).

#### Constant and hybrid funding

However, if the sponsor finds it difficult to verify the R&D investment level, then a constant funding function *F(C) = F*, independent of *C,* could be used. When this is so, for the particular case in which *F(C) = B = 1.2*, Table 3 summarizes the outcomes (see the supporting information S for further details).

**Table 3:** Comparing push, pull, mixed push-pull and PAYG mechanisms with constant funding *F(C) = 1.2.*

	Expected profit*				Comment
Scheme	C = 0	C = 0.2	C = 0.4	C = 0.6	
**Push**	1.2	1.15	1	0.85	No incentive to invest in R&D
**Pull**	0	0.55	0.6	0.67	Optimal to choose the maximum R&D level
**Mixed**	0.2	0.65	0.66	0.70	Maximum R&D is optimal and profit larger than with Pull scheme
**PAYG**	0.2	0.75	0.78	0.79	Maximum R&D is optimal and profit larger than with Pull and Mixed schemes

* The expected profit term includes the funding provided by the sponsor, whether or not it is actually spent on R&D by the firm. The net revenue *R* from a successful product is *0.3*. The probabilities of successful R&D investment for the firm (*C = 0*, *C = 0.2*, *C = 0.4* and *C = 0.6*) are, respectively, *0, 0.5, 0.66* and *0.85*.

In this case, the expected profit columns indicate that with constant funding, push schemes do not have the strength to alter the order of preference for R&D investment of the company in the case of no incentives (Table 1), and so the chosen level would still result in *C = 0*. However, pull incentives instead are now capable to reverse such order, inducing the company to choose spontaneously *C = 0.6*.

In summary with profit maximisation and constant funding, while firms in general would prefer push incentives, pull incentives appear to be more powerful at stimulating R&D investment. So, there could be a trade-off between enhancing the expected profit of the company and increasing success probability. Mixed push–pull incentive schemes, in which part of *F(C)* is given upfront but the rest only upon successful drug discovery can mitigate such tension. Indeed, as illustrated by Table 3, mixed schemes could still induce *C = 0.6* while increasing the expected profit of the company with respect to pull incentives; that is, mixed schemes could be economically more efficient. This is because, with respect to pull incentives, mixed schemes are better at sharing the risk of financial losses between the company and the sponsor. For this reason, whenever possible for the sponsor, an efficient way to separate the budget in the two push–pull components is to first identify the pull component sufficient to induce the desired R&D investment and then allocate the remaining sum to the push component.

This last observation suggests that incentive schemes in which risk could be further shared may have the potential to improve economic efficiency. Indeed, as shown in the expected profit column of Table 3, a type of push–pull incentive mechanism known as pay-as-you-go (PAYG), in which the available budget *B* can be separated into more than two components to incorporate intermediate, as well as final, goals set by the sponsor can achieve so. This could explain their increasingly frequent adoption.

It is worth noticing that unlike Table 2, push incentives in Table 3 would induce no R&D investment with expected profit maximisation but maximum investment with the non-negative profit goal.

## Discussion

The models presented in this paper are built on two main assumptions: first, that the level of investment made by the firm is based on expected profits maximization [15] and, second, that the probability of success increases with increasing levels of investment, but at decreasing rates. The possible limitations of such assumptions are discussed at length in the supporting information.

Determining the shape of the relationship between investment and the probability of success is also a key aspect for sponsors aiming to apply the points illustrated by this analysis, because the relative effectiveness of alternative incentive schemes is based on it. An estimation making use of available data, based on a recent study of the costs of the different phases for developing a new drug, is presented in the supplementary material. This analysis confirms that incentive schemes based on constant sums appear to be much less effective at inducing R&D investment by business companies, and/or more expensive for the sponsor, than simple linear incentive functions such as *F(C) = bC*. Indeed, in general, incentive programs can induce profit maximizing firms to invest in R&D if the funding does not add up as a constant to the firm expected profit.

## Materials and Methods

The paper is based on the analysis of a stylized economic model. So the only materials used are the data from Paul *et al* [16], in the supporting information S1, while methods are just economic and mathematical analysis.

## Supporting Information

Supporting Information S1 (DOCX)
