# Supplementary material for: R&D Incentives for Neglected Diseases
Source: PLoS One. 2012 Dec 19;7(12):e50835. doi: 10.1371/journal.pone.0050835 (PMC3526606; doi:10.1371/journal.pone.0050835)
Supplement: Supporting Information S1 — (DOCX) [file pone.0050835.s001.docx]

**Supporting Information S1**

**1 The Economics of Drug Discovery and Drug Delivery**

Assuming firms decisions to be driven by profitability considerations, the comparison of alternative incentive programmes stimulating R&D effort should not simply focus on the level of R&D expenditures, but on all the elements determining the expected profit level of a firm.

If $C$ indicates a company’s R&D investment level and $R$ the firm prospective profits (future revenues minus production and delivery costs) once a new drug is discovered and sold then a simple, convenient, way to visualize all such elements is illustrated by Table S.

The two stages of drug discovery and drug delivery are sequential but, of course, connected since the decision on how much to invest in R&D in the first stage would be fundamentally affected by $R$.

Therefore, in general, the higher $R$ the stronger the incentive to invest in R&D. The final decision however will also have to take into account the likelihood of success. If prospective profits are high but the success probability particularly low then R&D investments may be low.

Indeed, in the first stage, R&D expenditures affect two components of a company’s expected profits, along two opposite directions: (i) the total costs of the company and (ii) the drug discovery success probability $p\left( C \right)$*.* While, as costs, R&D expenditures reduce profits, they also typically enhance the probability of successful drug discovery, and hence the potential of enjoying future profits related to drug commercialization. The final choice on how much to invest in R&D would strike the balance between increasing the likelihood of success and increasing costs.

**2 The Expected Profit of a Company**

In this section we introduce the reference model of the analysis, a stylized representation of the firm behaviour. Though the goal of the paper is to identify the main underlying forces at work behind alternative R&D incentives, in Section 11 we shall also sketch how the model could be applied by sponsors. Successful drug discovery is inherently uncertain, and the firm returns fundamentally influenced by this. Success probability can be affected by the firm R&D effort; yet R&D investment is a necessary though not sufficient condition for successful discovery. As a consequence, once *C* is chosen, the firm profit $\Pi\left( R,C \right)$can take two possible values, according as to whether or not discovery is successful. If, with probability $(1-p\left( C \right))$, discovery fails then profits $\Pi\left( R,C \right)$will coincide with R&D losses, $-C$, while with successful discovery profits will be equal to $R-C.$ A more articulated version of this model could conceive “partial failure” outcomes, where even in case a new drug will not be discovered not all investment is lost. However, to simplify the exposition, without losing much generality, we shall consider only two outcomes.

The following scheme summarizes the point

$\Pi\left( R,C \right)=\left\{ \begin{aligned} R-C with probability p(C) \\ -C with probability (1-p\left( C \right)) \end{aligned} \right.$ (1)

To decide how much to invest in R&D in the first place it is standard to model the firm behaviour as maximizing its expected profit expression $E\Pi\left( R,C \right)$, the profit that “on average” would accrue to the company, defined as follows

$E\Pi\left( R;C \right)=p\left( C \right)\left( R-C \right)-\left( 1-p\left( C \right) \right)C=p\left( C \right)R-C$ (2)

The above expected profit expression is consistent with the previous intuition. What a firm expects to obtain are the future profits, weighted by the success probability, minus the R&D costs, which instead are afforded with certainty. Profits are weighted because they cannot be obtained for sure after R&D investments are made; therefore when success probability is low, even if perspective revenues $R$ are high, R&D investments could be discouraged.

When no external incentives are provided, based on (1) it is easy to see how to determine the four expected profit levels appearing in Table 1 of the main text, which below we further detail as Table S1

**3 The General Model**

In this section we elaborate on the more general version of the model, as formulated in expression (2), than the one in Table A1 which contemplates only a finite number of possible R&D investment levels. In particular, we assume *C* to be a continuous variable and *p(C)* a twice differentiable probability function, such that *p*$\left( 0 \right)=0, 0\leq p\left( C \right)\leq1$, $p^{'}\left( C \right)>0 \mathrm{and}p^{''}\left( C \right)<0$ to formalize the idea that a higher *C* increases the likelihood of discovery, though at decreasing rates. It is important to notice that from these assumptions does not follow that ${Lim}_{C\to\infty}p(C)=1$. For example, the probability function $p\left( C \right)=\frac{C}{2(1+C)}$ satisfies the above conditions but converges to $\frac{1}{2}$, suggesting that the discovery process could be so difficult that, even with a large amount of R&D investment, success probability cannot go beyond $\frac{1}{2}$. Below, and in the following sections, we shall further elaborate on this hypothesis. Note also that taken together the above assumptions imply that ${Lim}_{C\to\infty}p'\left( C \right)=0$, namely that when the R&D effort is already very high additional investments would basically leave unaltered the success probability.

Some comments are in order here.

In the analysis we assume $p\left( C \right)$ to be independent of the incentive scheme adopted by the sponsor, but there could be situations where timing of the financial aid, and not only size, could affect the performance. When this is so, the shape of $p\left( C \right)$ may vary across alternative funding programmes.

The function $p\left( C \right)$formalizes the difficulties intrinsic in the R&D activity and though the above assumptions seem quite general, there may be a number of exceptions to them. For instance decreasing rates from R&D investment level, $p^{''}\left( C \right)<0,$ could be valid up to a certain critical level of $C$after which increasing returns,$p^{''}\left( C \right)>0$, may prevail. Alternatively, returns could be initially increasing and decrease afterwards. Or else, the difficulty intrinsic to the discovery activity could be so high that after a certain threshold the function $p\left( C \right)$ will stop increasing in $C$, and be such that $p^{'}\left( C \right)=0$ and $p\left( C \right)<1$, for high enough values of $C$. Indeed, the recent debate on the productivity slowdown of the pharmaceutical industry could be considered as an important illustration of a non-increasing $p\left( C \right)$ as $C$ grows. Moreover, it may be that $p\left( C \right)=0$ up to a certain level of $C$, and start growing afterwards, to formalise the idea that for successful discovery at least a certain, minimal, threshold of positive investment in R&D is needed. Finally, modelling the development of a “second use” drug, that is a treatment building on an already existing compound it may be that $p\left( 0 \right)>0.$ Development costs in this case could be reduced, as the success probability function may shift upward with respect to when development is carried on from the very beginning, to formalize the idea that now drug registration may be easier, for any level of R&D investment.

Then, for any given *R* we assume the firm would choose $C$ to maximize $E\Pi\left( R;C \right).$If by $C(R)$ we indicate the R&D level maximizing (2) then it could either be $C\left( R \right)=0$ or $C\left( R \right)>0$.

Positive investments would satisfy the following first order, optimal, condition

$p^{'}\left( C(R) \right)= \frac{1}{R}$ (3)

which simply states that $C$ will be increased until the marginal benefit, induced by increasing the success probability is equal to the marginal cost. Implicit in (3) is the assumption that the optimal choice $C(R)$ strictly meets the firm budget. With no major loss of generality, such assumption will also be maintained throughout the analysis, since introducing budget constraints would not meaningfully alter the main findings and insights.

Whether or not $C\left( R \right)=0$ or $C\left( R \right)>0$ depends crucially on how “fast” the success probability function grows at low R&D investment levels. More specifically, given the assumptions on $p\left( C \right),$ if the R&D marginal expected benefit $p^{'}\left( 0 \right)R$ at $C=0$ exceeds its marginal cost, equal to $1$, then $C\left( R \right)>0$ otherwise $C\left( R \right)=0$. Indeed, positive expected profits could be obtained only if $p^{'}\left( 0 \right)R>1$.

Identifying the shape of $p\left( C \right)$ may not be an easy task and later, in Section 6, we shall discuss a possible way to do so in practice. Meanwhile, for a simple illustration of the previous point suppose, for example, that $\left( C \right)=\frac{C}{(a+C)}$ , with$a\geq0$, is the success probability function which satisfies all the above assumptions.

The parameter $a$ formalizes the intrinsic difficulty of discovery, which in the analysis is given. In particular $p\left( C \right)$ is equal to one when $a=0$ while $p(C)$ tends to zero as $a$ becomes large. Hence the larger the parameter value the more difficult is discovery. Moreover, if $a>0$ then $p\left( C \right)$gets close to one when $C$ is sufficiently large.

Finally, notice that $p\left( C \right)=\frac{C}{(a+C)}$is a version of the so called Tullock “contest success probability function”, widely used in Economics to model the winning probability of a subject (individual, firm etc.) competing with an opponent [16]. More specifically, suppose $x\geq0$ and $y\geq0$ are the effort levels of, respectively, subjects $A$ and $B$ competing against each other. Then in its standard version the Tullock winning probability function of subject $A$ would be $p_{A}\left( x,y \right)=\frac{x}{(x+y)}$ while for subject $B$ would be $p_{B}\left( x,y \right)=\frac{y}{(x+y)}$. Therefore, $p\left( C \right)=\frac{C}{(a+C)}$ is modelling the success probability of a firm “competing against nature”. Later on, when we apply the model to real data, the success probability function will be formalized differently.

With this success probability (2) becomes

$E\Pi\left( R;C \right)=\frac{C}{(a+C)}R-C$ = $\frac{C(R-a-C)}{(a+C)}$

Therefore $E\Pi\left( R;C \right)>0$, and so $C(R)>0$, requires $0<R-a$, namely $R>a$, which could also be obtained from the condition

$$p^{'}\left( 0 \right)R= \frac{1}{a}R>1$$

If, as in the example of the main text, $R=0.3$ then R&D investments would be profitable for the firm if $a<0.3$, namely if discovery is sufficiently simple. When $R>a$ then (3) becomes

$$\frac{a}{{(a+C)}^{2}}= \frac{1}{R}$$

so that the optimal level of R&D investment is $C(R)= \sqrt{aR}-a$ and the maximum profit given by $E\Pi\left( R;C(R) \right)={(\sqrt{R}-\sqrt{a)}}^{2}$. Hence, in this case, $C(R)$ has a very simple and interesting interpretation: the larger the difference between $R$ and $a$ the higher the R&D investment level. Notice however that $C(R)$, viewed as a function of $a,$ would be maximum at $a=\frac{R}{4},$ rather than at its boundaries $a=0$ and $a=R$. Intuitively, there is little or no incentive to invest in R&D when discovery is *too easy*, namely $a=0$ or *too difficult*, $a\geq R$, with respect to the prospective profits. As a consequence, since the highest possible R&D investment level would be equal to $\frac{R}{4}$, then the firm would never invest more than 25% of the prospective profits. With $R=0,3$ the company would invest at most $\frac{0,3}{4}=0,075,$ and in so doing enjoy an expected profit of the same amount.

**4 Comparing R&D Incentives**

This section provides the needed, supplementary information, to explain the expected payoff values associated to the different incentive schemes appearing in Table 2 and Table 3 of the main text, as well as to discuss incentive programmes within the more general version (2) of the model.

Comparison of alternative schemes will be done assuming that funds are represented by a monetary amount $F(C)$, with $F\left( C \right)\geq0$ and $F^{'}\left( C \right)\geq0$ provided by the sponsor. The general form of such funding function is given by $F\left( C \right)=F+f(C)$, where $F\geq0$ is the constant part of the funds and $f\left( C \right)\geq0$ the variable part, which depends on the firm R&D effort. The $f(C)$ function specifies the co-funding part of the sponsor. A simple specification of such function is the linear funding $F\left( C \right)=F+bC$, where $b\geq0$. Finally, throughout this section we assume $p^{'}\left( 0 \right)R<1$, in the example $a>R$, namely that without outside funding the firm will not invest in R&D and that, moreover, the sponsor is endowed with a budget $B$, so that $F\left( C \right)=F+f(C)\leq B$, for any investment level $C$ made by the company.

**5 R&D Investments with Unconditional Push Incentives**

Suppose the firm receives an up-front sum $F(C)$ by the sponsor; the sum is provided unconditionally, that is without the sponsor requiring the firm to invest $F(C)$ in R&D. Then (1) takes the following form

$E\Pi_{push}(R;C;F(C))=\left\{ \begin{aligned} R+F(C)-C with probability p(C) \\ F\left( C \right)-C with probability (1-p\left( C \right)) \end{aligned} \right.$ (4)

so that (2) becomes

$E\Pi_{push}\left( R;C;F(C) \right)=p\left( C \right)\left( R-C+F(C) \right)-\left( 1-p\left( C \right) \right)\left( C-F(C) \right)=p\left( C \right)R-C+F(C)$ (5)

hence

$E\Pi_{push}\left( R;C;F(C) \right)=E\Pi\left( R;C \right)+F(C)$ (6)

showing that the presence of $F(C)$ shifts upward by the same amount the firm expected profit, with respect to when $F(C)$ is not available. Since $E\Pi\left( R;C=0 \right)=0$ availability of $F(C)\geq0$ would in general imply positive expected profits. A further main difference with respect to (2) is that when $F^{'}\left( C \right)\geq1$ expression (6) is increasing for all $C\geq0,$ which means that the firm would have an incentive to invest as much as possible in R&D. But since $F^{'}\left( C \right)\geq1$ implies that the sponsor co-funding is at least as large as the funding effort $C$, this means that in this case push incentives induce the highest possible R&D investment level.

Therefore, depending upon the shape of $F\left( C \right)$ conditions for positive R&D investment levels may differ from (3), since $E\Pi\left( R;C \right)$ and $E\Pi_{push}\left( R;C;F(C) \right)$ are in general maximized by different values of $C$. However, when $F\left( C \right)=F$, and so $F^{'}\left( C \right)=0$, then it is clear that (3) and (6) are maximized by the same value of $C$.

To summarize, the above considerations suggest that the strength of push incentives as drivers to stimulate R&D effort appear to be sensitive to how $F\left( C \right)$ grows, namely to $F'\left( C \right)$. In particular, the larger $F'\left( C \right)$ the higher the firm R&D effort. Analogously, the lower $F'\left( C \right)$ the less effective are push incentives in inducing firms to change spontaneously their most preferred R&D investment levels.

If ${0<F}^{'}\left( C \right)<1$ and $F^{''}\left( C \right)<0$ then $E\Pi_{push}\left( R;C;F(C) \right)$ is concave and has a unique maximum. Hence the optimal R&D investment level $C_{push}$ can be obtained by solving the first order condition

$p^{'}\left( C_{push} \right)= \frac{1-F^{'}\left( C_{push} \right)}{R}$ (7)

which clearly implies that $C_{push}\geq C(R)$, where equality obtains when $F^{'}\left( C \right)=0$. For instance, if in the previous example we define $F\left( C \right)=F+bC$, with $0<b<1$, then solving (7) would give rise to $C_{push}= \sqrt{\frac{aR}{1-b}}-a > C(R)$.

We conclude this section with Table S2 below, which based on (4) clarifies how the firm payoff values with push incentives, in Table 2 and Table 3 of the main text, are obtained.

**6 R&D Investments with Conditional Push Incentives**

It may be interesting to discuss briefly what happens if $F\left( C \right)$ is provided by the sponsor only conditionally upon the firm investing it in R&D, which the sponsor can verify. In this case, still assuming $0<F'\left( C \right)<1$ and $F''\left( C \right)<0$ expression (5) becomes

${E\Pi}_{push}^{*}\left( R;C;F(C) \right)=p\left( C+F(C) \right)R-C$(8)

and condition (7) for the total, optimal, R&D level $C_{push}^{*}+F(C_{push}^{*})$, would now be

$p^{'}\left( C_{push}^{*}+F(C_{push}^{*}) \right)= \frac{1}{R(1+F^{'}(C_{push}^{*})}$ (9)

It is immediate to verify that $C_{push}^{*}+F(C_{push}^{*})<$ $C_{push}.$ Thus, perhaps somewhat counter-intuitively, if the sponsor wishes the firm to invest the donated sum in R&D then this would induce an overall research effort lower than when the funds are donated unconditionally. Intuitively this is because, when funds are conditional, $F(C)$ can affect the firm expected profit only indirectly, through the likelihood of discovery and so through the R&D overall costs, however without increasing the prospective profits, which is instead what happens when funds are given unconditionally.

Continuing with the previous example, the optimal condition (9) would now give rise to

$C_{push}^{*}+F(C_{push}^{*})= \sqrt{aR(1+b)}-a$ < $\sqrt{\frac{aR}{1-b}}-a= C_{push}$.

**7 R&D Investments with Pull Incentives**

Suppose now that the company receives funds $F(C)$from the sponsor, but only conditionally upon drug discovery having been successful. Hence, in this case, $F(C)$ would operate as a pull incentive. Then (1) now becomes

$E\Pi_{pull}(R;C;F(C))=\left\{ \begin{aligned} R-C+F(C) with probability p(C) \\ -C with probability (1-p\left( C \right)) \end{aligned} \right.$ (10)

while (2) transforms into

$E\Pi_{pull}\left( R;C;F(C) \right)=p\left( C \right)\left( R-C+F(C) \right)- \left( 1-p\left( C \right) \right)C=p\left( C \right)(R+F(C))-C$ (11)

and so

$E\Pi_{pull}\left( R;C;F(C) \right)=E\Pi\left( R;C \right)+p\left( C \right)F\left( C \right)=E\Pi_{push}\left( R;C;F(C) \right)- \left( 1-p\left( C \right) \right)F\left( C \right)$ (12)

showing that, for any $C,$ it is $E\Pi_{pull}\left( R;C;F(C) \right)\leq$ $E\Pi_{push}\left( R;C;F(C) \right)$. Indeed, from (11) notice also that pull mechanisms with funding given by $F(C)$ are equivalent to push mechanisms with funding given by $p(C)F(C)$.

Yet, as we are going to discuss below, from the previous inequality is not obvious which of the two programs would induce a higher R&D effort by the firm.

Still assuming ${0<F}^{'}\left( C \right)<1$ and that $E\Pi_{pull}\left( R;C;F(C) \right)$ is concave and has a unique maximum, the optimal R&D investment level with pull incentives$C_{pull}$ is obtained by solving the following condition

$p^{'}\left( C_{pull} \right)= \frac{1-[p( C_{pull})F\left( C_{pull} \right)]'}{R}$ (13)

Whether or not it is $C_{push}>C_{pull}$ can be seen by considering (12). Indeed, given the above assumptions we only need to check if at $C=C_{push}$ the function $E\Pi_{pull}\left( R;C;F(C) \right)$ is increasing or decreasing. In the former case it would be $C_{pull}>C_{push}$ while in the latter $C_{pull}<$ $C_{push}$. So, differentiating (12) we obtain

$\frac{dE\Pi_{pull}\left( R;C;F(C) \right)}{dC}$ = $\frac{dE\Pi_{push}\left( R;C;F(C) \right)}{dC}- F^{'}\left( C \right)+[p\left( C \right)F\left( C \right)]'$

which at ${C=C}_{push}$ becomes $- F^{'}\left( C_{push} \right)+[p\left( C_{push} \right)F\left( C_{push} \right)]'$, since by definition $C_{push}$ solves the equation $\frac{dE\Pi_{push}\left( R;C;F(C) \right)}{dC}=0$. Therefore if

$- F^{'}\left( C_{push} \right)+\left[ p\left( C_{push} \right)F\left( C_{push} \right) \right]^{'}>0$ (14)

then $C_{push}<$ $C_{pull}$, and viceversa.

A way to characterize condition (14) is by rearranging it as follows

$$\frac{{}_{F}( C_{push})}{{}_{p}( C_{push})}<\frac{p( C_{push})}{1-p(C_{push})} (15)$$

where on the left hand side of (15) the expressions ${}_{F}\left( C \right)= \frac{F'\left( C \right)C}{F(C)}$ and ${}_{p}\left( C \right)= \frac{p'\left( C \right)C}{p(C)}$ indicate, respectively, the elasticity of functions $F(C)$ and $p(C)$ calculated at ${C=C}_{push}$ while the right hand side of (15) represents the “odds” ratio, namely the ratio between success and failure probabilities, still calculated at ${C=C}_{push}$.

Since ${}_{F}\left( C \right)$ expresses the percentage change of $F(C)$ relative to the percentage variation of $C$, and analogously for ${}_{p}\left( C \right)$, then (15) simply says that pull incentives stimulate higher R&D effort than push incentives if $p(C)$ is sufficiently more reactive than $F(C)$ at $C_{push}.$

Condition (15) clarifies that which, between push or pull, is a stronger incentive depends upon the shape of $F(C)$ and $p\left( C \right).$ But while the form of $p\left( C \right)$ could be considered as given, at least for a period of time, and cannot be chosen by the sponsor, this is not so with $F\left( C \right)$ which indeed is selected by the donator.

Therefore, though not an easy task, the sponsor should try to collect as much information as possible on $p\left( C \right)$ and, based on it, define $F\left( C \right)$ in such a way as to induce the highest R&D effort by the firm. More specifically, once the general form of $F\left( C \right)$ is chosen, the sponsor should decide between push and pull by comparing the best outcomes of the two incentive programmes.

To illustrate the above point consider again the previous example, still assuming the linear funding function $F\left( C \right)=F+bC$, with $F\geq0$ and $0\leq b<1$. Then ${}_{F}\left( C \right)= \frac{bC}{F+bC}$ while ${}_{p}\left( C \right)= \frac{a}{a+C}=(1-p\left( C \right))$. Therefore, for a generic $C$ condition (15) now becomes

$$\frac{bC}{F+bC}< \frac{C}{a+C}$$

leading to the following inequality

$a<\frac{F}{b}$ (16)

which is independent of $C$(and also of $R)$. Thus, for given $F\left( C \right),$ in the example pull incentives would be preferable to push schemes if the ratio $\frac{F}{b}$, indicating the relative strength of the fixed and variable components of $F\left( C \right),$ is sufficiently high. Condition (16) could also be obtained by noticing that (13) implies

$C_{pull}= \sqrt{\frac{a(R+F-ab)}{1-b}}-a$ (17)

and so $C_{pull}\geq C_{push}$ if $\frac{a(R+F-ab)}{1-b} \geq\frac{aR}{1-b}$which is satisfied when (16) holds.

Using incentives schemes $F\left( C \right)$ that depend on $C$ requires perfect verifiability by the sponsor of the firm R&D investment level and knowledge of $p\left( C \right)$. Hence, if the sponsor could not reliably ascertain how much the firm invested, as well as $p\left( C \right),$ proper implementation of the scheme may be prevented.

For this reason, even when push programs may have a stronger potential for stimulating the firm R&D investments, if $C$ is difficult to monitor then the sponsor may prefer to donate a constant sum $F\left( C \right)=F$, in which case pull incentives would be preferable for the sponsor to push incentives.

But the analysis in the main text shows that in case the sponsor uses constant funding then the following trade-off emerges. If pull incentives stimulate a higher R&D level by the firm push schemes however provide a higher expected payoff to the firm. More explicitly, to stimulate higher investments the sponsor has to penalize the firm. In the next section we shall discuss how this trade-off could be, at least partially, mitigated by mixed push-pull incentives.

However, prior to doing so in Table S3 below we clarify how the expected profit levels with pull incentives, appearing in Table 2 and Table 3 of the main text, are determined

**8 R&D Investments with Mixed, Push-Pull, Incentives**

To deal with the above mentioned trade-off, in case of constant funding, it is natural to think of mixing, combining, the two programs. To see how suppose that the amount $F\left( C \right)=F$ is split into two components $F=F_{push}+ F_{pull}$, with $F_{push}$ being provided as an upfront sum and $F_{pull}$ only upon successful drug discovery.

Then (1) would now take the form

${E\Pi}_{mixed}(R;C;F)=\left\{ \begin{aligned} R-C+F with probability p(C) \\ -C+F_{push} with probability (1-p\left( C \right)) \end{aligned} \right.$ (18)

while (2) becomes

${E\Pi}_{mixed}\left( R;C;F \right)=p\left( C \right)\left( R-C+F \right)-\left( 1-p\left( C \right) \right)(C-F_{push})$ (19)

and so

${E\Pi}_{mixed}\left( R;C;F \right)=p\left( C \right)\left( R+F_{pull} \right)-C+F_{push}={E\Pi}_{pull}\left( R;C;F \right)+(1-p\left( C \right))F_{push}$ (20)

Since $(1-p\left( C \right))F_{push}\geq0$ the above expression suggests that with mixed incentives firms could have the potential to increase the expected profit with respect to pull programs. According to the previous analysis, the condition for positive R&D investment now becomes $p^{'}\left( 0 \right)(R+F_{pull})>1$, more demanding than with pure pull, but less than with pure push, incentives.

If investments are positive then their optimal level $C_{mixed}$will typically be determined by the condition

$p^{'}\left( C_{mixed} \right)= \frac{1}{(R+F_{pull})}$ (21)

which indicates that in general $C_{mixed}<C_{pull}$. So, in general, as compared to pull programs mixed incentives tend to be more preferable for firms and less so for the sponsor.

However, if the sponsor is interested in reaching a specified level of R&D investment, rather than the highest possible effort, then mixed programs could be more efficient than pull incentives, as they might increase the firm expected profit while allowing the sponsor to achieve his own goal.

To illustrate the point consider again $p\left( C \right)=\frac{C}{(a+C)}$, assuming $F=1.2, a=$1 and that for the sponsor the desired level $C^{d}$of R&D effort is $C^{d}=0.2$. With pure pull incentives the firm would find it optimal to invest $C_{pull}= \sqrt{a\left( R+F \right)}-a= \sqrt{1.5}-1=0.22>$ $C^{d}$. Then, clearly the sponsor would not need to use the whole budget $B=F=1.2$ as a pull incentive, but just what is required to induce $C_{pull}=C^{d}=0.2$, employing the residual as push component. It is possible to determine the pull component by solving for $F_{pull}$ the following equation

$$C^{d}= \sqrt{a(R+F_{pull})}-a$$

which in our numerical example provides

$$F_{pull}={(1.2)}^{2}-0.3= 1.14$$

and so $F_{push}=0.06$. This way the sponsor could still obtain $C^{d}=0.2$, while increasing the firm expected profit level.

We conclude this section with Table S4, illustrating how the firm expected profits in Table 3 of the main text are obtained.

**9 R&D Investments with Pay-As-You-Go (PAYG) Incentives**

If simple mixing of push and pull incentives can prove to be a more efficient mechanism than pull schemes then it is natural to think that improvement in efficiency may be obtained by further articulating the mixed program. A way to do so is PAYG, a scheme where incentives can be provided ad interim, upon achieving intermediate goals specified by the sponsor, as well as up front and when the drug is eventually developed.

Still considering constant funding, a simplest PAYG scheme would consist in introducing an additional intermediate goal specified by the sponsor which, if successfully reached by the firm, would be rewarded with the sum $F_{interim}$. So the time span between the beginning and the end of the R&D activity can now be divided into two phases: phase 1 which goes from the beginning of the activity until the intermediate goal is reached, and phase 2 from then until the end. Therefore, with respect to the previous section $F$ too would now be subdivided as $F=F_{push}+$ $F_{interim}{+F}_{pull}$, where the $F_{interim}$ component plays the role of both a pull incentive for phase 1 and of a push incentive for phase 2.

Moreover, the presence of two phases will also separate the R&D effort into two components $C=C_{1}{+C}_{2}$, where $C_{1}$ is the R&D investment in the first phase and $C_{2}$ in the second phase. Finally, by $h\left( C_{1} \right)$ we indicate the success probability for the first phase and by $g(C_{2})$ for the second phase, conditionally upon the first phase having been successful.

Because of the presence of an intermediate goal, the firm now has to take a more elaborate decision. Indeed, as well as to evaluate whether it is profitable to invest in both phases or not at all, it also has to decide if investing *only* in the first phase could be a profitable option. This suggests that the sponsor has to define the two phases, and related incentive components, in such a way that the firm would find it profitable to invest in both of them, rather than stop investing after the first phase without completing the project.

If the firm invests in both phases then its expected payoff is given by

${E\Pi}_{PAYG}\left( R;C;F \right)=h\left( C_{1} \right)g\left( C_{2} \right)\left( R+F_{pull} \right)+h\left( C_{1} \right)\left( F_{interim}-C_{2} \right)+ (F_{push}-C_{1})$ (22)

In this case, if $h\left( C_{1} \right)$ and $g\left( C_{2} \right)$enjoy the same properties as $p(C)$, the optimality condition (when pertinent) for $C_{1}$is

$h^{'}\left( C_{1} \right)= \frac{1}{g\left( C_{2} \right)\left( R+F_{pull} \right)+\left( F_{interim}-C_{2} \right)}$ (23)

and for $C_{2}$

$g^{'}\left( C_{2} \right)= \frac{1}{\left( R+F_{pull} \right)}$ (24)

which, reflecting the forward looking recursive nature of (23), shows that while phase 2 investments do not depend on phase 1 research effort, $C_{1}$ instead depends also on $C_{2}.$

If the firm invests only in phase 1 its expected profit would instead be

${E\Pi}_{PAYG}^{F}\left( R;C;F \right)=h\left( C_{1} \right) F_{interim}+ F_{push}-C_{1}$ (25)

and the optimality condition for $C_{1}$becomes

$h^{'}\left( C_{1} \right)= \frac{1}{F_{interim}}$ (26)

showing that the level of phase 1 investment changes according as to whether the firm invests in both phases.

If PAYG is designed properly then the sponsor may enjoy a higher overall R&D effort with respect to mixed push-pull programmes and, under appropriate conditions on the success probability functions and the scheme of rewards, at the same time also improve the firm expected payoff.

Though this doesn’t necessarily occur, the following numerical example illustrates how it might happen.

Consider PAYG first and suppose $h\left( C_{1} \right)= \frac{C_{1}}{(0.2+C_{1})}$ and $g\left( C_{2} \right)= \frac{C_{2}}{(0.3+C_{2})}$ , $F_{push}=0.2$, $F_{interim}=0.5$, $F_{pull}=0.5$ and $R=0.3.$Then if the firm invests in both phases from(23)-(24) it obtains ${E\Pi}_{PAYG}\left( 0.3;C;1.2 \right)=0.31$, with $C_{1}=0.15$, $C_{2}=0.19,$so that the overall research effort would be $C_{PAYG}=C_{1}+C_{2}=0.34$. Moreover, if the firm contemplates investing only in phase 1 from (25)-(26) it would obtain ${E\Pi}_{PAYG}\left( 0.3;C;1.2 \right)=0.26$, with $C_{1}=0.12$, and so would prefer investing in both phases.

Consider now a mixed push-pull program with $\left( C \right)= \frac{C}{0.5+C}$ , $F_{push}=0.2$ and $F_{pull}=1$.

In this case ${E\Pi}_{mixed}\left( 0.3;C;1.2 \right)=0.3$ and $C_{mixed}=0.3$, so that with the PAYG scheme the firm is better off and the sponsor induces a higher overall level of R&D investments. Yet, the probability of final discovery in the PAYG scheme, $\left( \frac{0.15}{0.2+0.15} \right)\left( \frac{0.19}{0.3+0.19} \right)=0.17$ will be much lower than the probability of drug discovery in the mixed scheme $\frac{0.3}{0.5+0.3}=0.38$.

This consideration suggests that for the probability of discovery to be higher with PAYG than with mixed programs it may not be enough to have higher overall R&D investments, as they need to be sufficiently larger. In the example this could happen for instance when $h\left( C_{1} \right)= \frac{C_{1}}{(0.1+C_{1})}$ and $g\left( C_{2} \right)= \frac{C_{2}}{(0.1+C_{2})}$, namely if the sponsor was able to express the entire drug discovery process as the combination of two phases, each with much higher chances of success. Indeed, in this case it would be$h\left( C_{1} \right)=0.65=g\left( C_{2} \right)$and the success probability equal to ${0.65}^{2}=0.42>0.38$. Moreover, such probability values will also maximize the firm expected payoff as compared to investing only in phase 1, and to the mixed push-pull scheme.

Finally, Table S5 clarifies how the values of ${E\Pi}_{PAYG}\left( R;C;F \right)$ in Table 3 of the main text were determined. As above we assume $F_{push}=0.2$, $F_{interim}=0.5$, $F_{pull}=0.5$. Moreover the example is an illustration of how PAYG can increase its efficiency in a more general case where, unlike $g\left( C_{2} \right),$ $h\left( C_{1} \right)$ increases more than proportionally with $C_{1}$.

We conclude this section observing that though partly inspired by data characterizing the pharmaceutical industry [15], the parameter values in Tables S1-S5 have been mainly chosen for illustrative purposes. Indeed, a more articulated discussion on how the model can be applied to real data is postponed until Section 11.

**10 Merits and Limitations of the Analysis**

We think the analysis has a number of merits, but also limitations, that we now want to discuss.

To our knowledge, this is the first attempt to compare alternative R&D incentive schemes for neglected diseases within a formal framework. For this reason, under the assumptions of the model the work provides concise indications and guidelines, clarifying which incentive schemes could be preferable for the sponsor, as well as efficient. Indeed our findings are consistent with the empirical evidence, and so represent a possible explanation that Mixed Push-Pull schemes, notably Pay-As-You-Go, are more and more frequently used in practice. Indeed, while pull incentives are more preferable for the sponsor, push schemes are more desirable for the company. Therefore, it is not surprising that a balanced mixture of the two would appear as the most effective device to stimulate R&D effort, at the same time inducing efficiency.

The possible limitations of any model are given by the assumptions behind the model itself. Clearly, the more realistic the hypothesis the more reliable are the model predictions.

Among others, the paper is based on the two main assumptions of (i) profit maximization and the (ii) success probability $p(C)$ wich is increasing ($p^{'}\left( C \right)>0$) at decreasing rates ($p^{''}\left( C \right)<0$), that we now want to elaborate upon.

In what follows we focus on the assumption of expected profit maximization, postponing until the next Section the discussion on the shape of the success probability. Profit maximization is a standard and widely accepted way to represent the economic goal of a business firm, reflecting the idea that shareholders (firm owners) want to maximize their values. However, in certain circumstances the prevailing goal of a company may be different. In particular, this seems to be the case in the current period, where pharmaceutical firms appear to be more concerned about productivity than about profits [15].

Productivity becomes a priority when companies take the rate of return from R&D investments as reference to set their economic goals. Such shift in the goal may not be trivial, since focusing on productivity, rather than on profits, can have important bearings on the R&D investment level.

To gain some insights on how things may change let $\Gamma\left( C \right)$, defined as

$$\Gamma(C)=\frac{p\left( C \right)R-C}{C}$$

be the standard indicator of productivity, given by the ratio between expected profits and R&D costs, namely by the “average expected profits”. Then

$$\Gamma^{'}\left( C \right)=\frac{R\left[ p^{'}(C)C-p\left( C \right) \right]}{C^{2}}$$

which is greater than zero if ${}_{p}(C)=\frac{p^{'}(C)C}{p(C)}>1,$ namely if the elasticity of the success probability function is larger than one. This implies that, unlike what happens when expected profits are of concern, now perspective profits $R$ play no role in deciding whether or not to invest in R&D, but investments will only depend upon the shape of the success probability $p\left( C \right)$. It is simple to verify that if $p^{''}\left( C \right)<0$ then ${}_{p}\left( C \right)=\frac{p^{'}\left( C \right)C}{p\left( C \right)}<1$. Consequently, $\Gamma^{'}\left( C \right)<0$ and no R&D investment would be made in this case. For example this would be if, as before, $p\left( C \right)=\frac{C}{a+C}$ since ${}_{p}\left( C \right)=\frac{a}{a+C}<1$. Intuitively, concern for productivity is much more sensitive to the likelihood of success than concern for expected profits.

We now discuss if, and how, things may change with incentives. Consider first push incentives $F\left( C \right)=F$, with only a fixed component. In this case

$$\Gamma_{push}\left( C \right)=\frac{p\left( C \right)R-C+F}{C}$$

and so

$$\Gamma_{push}^{'}(C)= \frac{[p^{'}\left( C \right)R-1]C-[p\left( C \right)R-C+F]}{C^{2}}=\frac{\left[ p^{'}\left( C \right)C-p\left( C \right) \right]R-F}{C^{2}}$$

showing that now the condition for productivity to be increasing in $C$

$${}_{p}\left( C \right)>1+\frac{F}{p(C)R}$$

depends upon both $F$ and $R$ and that, rather intuitively, since $\frac{F}{p(C)R}>0$ the condition is more demanding on ${}_{p}\left( C \right)$ than with no incentives. Therefore push incentives, with fixed component only $F\left( C \right)=F$ may even discourage R&D investments, when the success probability function is such that

$${1<}_{p}\left( C \right)<1+\frac{F}{p(C)R}$$

and in so doing now represent a weaker driver for investing in R&D, as compared to when firms maximise their profits.

Moreover alternative goals, to expected profit maximization, could embody some more explicit, and deeper, concern for the risk of unsuccessful discovery and for losing the invested resources. One first possibility for a firm could be

$${Max}_{C} E\Pi\left( R;C \right)=p\left( C \right)R-C$$

however given the constraint that $p\left( C \right)\geq p^{*}$, namely that the success probability would be at least as high as a pre-specified threshold level $p^{*}$. In this case the optimal R&D investment level would either solve the equation $p\left( C \right)=p^{*}$ or maximize the profit, depending upon which of the two would be larger.

Alternatively, the goal could be

$${Max}_{C} \alpha E\Pi\left( R;C \right)+\beta p\left( C \right)=p\left( C \right)\left( R\alpha+\beta\right)-C$$

namely maximizing a linear combination of the expected profits and the success probability, where $\alpha$ and $\beta$ are the (non-negative) weights formalizing the firm concern for, respectively, expected profits and success probability. The first order condition

$p^{'}\left( C \right)= \frac{1}{\left( R\alpha+\beta\right)}$

maintains the same structure as $(3)$, entailing a larger R&D investment level if $\left( R\alpha+\beta\right)>R$, as when concern for $p(C)$ is high enough.

A further, more common, way to formalize concern for risk could be through the mean-variance approach as follows

$${Max}_{C} \alpha E\Pi\left( R;C \right)-\beta Var\Pi\left( R;C \right) (27)$$

where

$$Var\Pi\left( R;C \right)=R^{2}p\left( C \right)(1-p\left( C \right))$$

is the variance of the profits. In the simple case with $\alpha=1=\beta$ the first derivative of $\left( 27 \right)$ is given by

$$p^{'}\left( C \right)R-p^{'}\left( C \right)R^{2}+{2p}^{'}\left( C \right)p\left( C \right)R^{2}-1 (28)$$

which at $C=C\left( R \right)>0$, solving $(3)$, becomes $-p^{'}\left( C\left( R \right) \right)R^{2}+{2p}^{'}\left( C\left( R \right) \right)p\left( C\left( R \right) \right)R^{2}$ that is positive for $p(C\left( R \right))>\frac{1}{2}$. This condition suggests an interesting characterization for the optimal $C$, in this case, to be larger than when only profits are maximized. Intuitively, if at the profit maximizing R&D level the success probability is higher than $0.5$ then $R$ must be large enough, and concern for risk induces to invest more than $C\left( R \right),$ to further increase the chance of success for such an interesting outcome.

Moreover, because a number of organizations developing drugs for neglected diseases would be not-for-profit, then their goal could simply be to maximize the success probability, that is to invest as much as possible in R&D, provided profits are non-negative. In this case since, as we have seen previously, $E\Pi_{pull}\left( R;C;F(C) \right)\leq$ $E\Pi_{push}\left( R;C;F(C) \right)$ then a push mechanism will always be preferable to a pull, as it would induce at least as high R&D investments for any funding function. But of course, this is when “moral hazard” would not be an issue for the sponsor, that is when the institution developing the new drug would not behave opportunistically upon receiving the funds. Alternatively, as we assumed for most of the paper, funds could be used by the developer in its best interest which may not lead to the most desirable R&D investment level for the donator.

Finally, firms can choose the minimum $C$ solving $E\Pi\left( R;C \right)\geq\Pi^{*}$, that is a pre-specified, reasonable, expected profit level which may be lower than the maximum one. Under our assumptions, this would lead to decrease the R&D investment level.

**11 How to Make Use of the Analysis**

We conclude the supplementary material by sketching how sponsors could make use of the analysis to pursue their goals.

An important element for the sponsor to determine is the success probability of the firm $p(C)$, since the relative effectiveness of alternative incentive schemes is based on its shape$.$ This may be estimated by making use of available data. Particularly, in what follows, as an example we shall refer to [16] as well as to other contributions [18], discussing how to challenge the recent decrease in productivity faced by the pharmaceutical industry.

Table S6 below summarizes the relevant probabilities in Fig 2 of [16]. More specifically for each R&D phase, and each single compound, the first (top) row contains the transition probabilities from one phase to the next while the second row the associated attrition rate. Accordingly, the third row indicates the probability for a compound in a given phase to be successfully launched while the last, fourth, row the complement to the launch probability, representing the variance with which the number of successful compounds deviates from one, the expected number of launched molecules. Consistently with the intuition, even if on average the number of compounds in each phase guarantees one launch, the variance around one such expected launch reduces for phases nearer to final submission.

|  |  |  |  |  |  |  |  |  |  |
| --- | --- | --- | --- | --- | --- | --- | --- | --- | --- |

According to Fig 2 of [16] the overall out-of-pocket cost, in millions, for launching one molecule is about $\$873$. Therefore, investing less than $\$873$ would lower the success probability by decreasing the expected number of compounds at the submission-to-launch phase. This consideration is the starting point for our estimate of the success probability $p(C)$.

Indeed, in Table S7 below we define $C=\$873$to be $100\%$ investment in R&D to obtain on average one launch, with $1,1$ being the associated number of compounds at the submission-to-launch phase. Then, $p\left( C=\$873 \right)=0,91$ and a first, rather simple, attempt to reconstruct the function $p(C)$ could be to estimate its value at some investment levels $0<C<873$.

In particular, below we consider ten R&D investment levels $C=\frac{873i}{10}$, with $i=1,..,10,$assuming that the corresponding ten number of compounds at submission-to-launch phase would be determined proportionally by $\frac{1,1i}{10}$, which we use to calculate the success probability for such lower than $$873$levels of financial effort. A finer estimation of the function could be made by considering a higher (than ten) number of points.

Before we exemplified the analysis through the success probability function $p\left( C \right)=\frac{C}{a+C}$. However, with reference to the data in Table S6, in what follows we develop the discussion by seeing the R&D expenditure $C$ as the total investment made in a certain number of compounds, assuming that compounds can reach launch independently of each other according to the transition (as well as related success) probabilities summarized by Table S6. As we shall see, this will give rise to a success probability function different from the one used previously.

Since $0,91$is the transition probability from submission-to-launch to registration, the success probability with $\frac{1,1i}{10}$compounds in that phase can be estimated, as the probability of obtaining at least one launch, as follows

$p\left( C=\frac{873i}{10} \right)=1-\left( 1-0,91 \right)^{\frac{1,1i}{10}}$ with $i=1,\ldots.,10$

The relevant calculations are summarized in Table S7 below. Notice that the row containing the average success probability $\frac{p(C)}{C}$, being decreasing in $C$, indicates that the estimated $p(C)$ is increasing, though at decreasing rates, as we have been assuming in the paper.

Based on the above estimates, the next Table S8 summarizes some main findings of the analysis without incentives. Henceforth, asterisks in the Tables identify the maximum expected profit. In particular, still expressed in million dollars, Table S8 considers three levels of perspective profits. The first, $R=\$375,$ is a threshold below which the lowest R&D investment would be optimal. The second $R=\$1000$ represents an “intermediate” perspective profit level for which there is positive R&D investment, $C$ about $$350$, though less than half of the maximum $C=\$873$. Finally, $R=\$4100$ is a threshold level above which it is optimal for the company to choose $C=\$873$.

Therefore our findings suggest that, to induce the highest R&D investment, incentives should act “as strong drivers as” perspective profits $R=\$4100$.

In the following Table S9 we consider the lower threshold level $R=\$375,$namely an initial situation where no or low investment is made, and focus the discussion on how push and pull incentives compare in stimulating R&D effort. Based on the Table, we first observe that with constant incentives $F=\$1000$ while push schemes still induce the lowest $C=\$87,3$ R&D investment level, pull incentives are able to drive up to half of $$873, C=\$436$, R&D investments. In the remaining rows we compare the two schemes when the incentive has no fixed component, namely $F\left( C \right)=bC$. In the table we consider two critical values for $b$. The value $b=0,81$, is the smallest value inducing the highest $C=\$873$ with pull incentives. In this case, the sponsor could stimulate the maximum R&D effort by the firm spending about $\left( 0,81 \right)\$873=\$707,1$, roughly $\$165$ less than what the firm spends. However, with the same $b=0,81$ push incentives would induce much lower investments, about $\$611$. Indeed, for push incentives to induce the maximum investment it would have to be at least $b=0,91$, requiring the sponsor to spend about $\$794$. Therefore, as compared to pull schemes, to induce the highest $C=\$873$, with push incentives the sponsor would need to increase his donation by about $\$183.$ Therefore, when $R=\$375$ with pull schemes an incentive function $F\left( C \right)=0,81C$ would have the “same strength”, in terms of inducing the maximum R&D effort, as the constant sum $F=\$4100-\$375=\$3725$, allowing the sponsor to save about $\$3725-\$707=\$3018$.

We conclude this section by renewing the observation that incentive schemes based on constant sums appear to be much less effective to induce R&D effort, and/or more expensive for the sponsor, than simple linear incentives functions such as $F\left( C \right)=bC$

**Tables Section**

**Table S**

|  | **First Stage** | |
| --- | --- | --- |
|  | *R&D Costs* | *Success Probability* |
| **Drug Discovery** | $C$ | $p(C)$ |
|  | **Second Stage** | |
|  | *Perspective Profits* | |
| **Drug Delivery** | $R$ | |

**Table S1**

**The Firm Expected Profit with No Incentives**

| **C** | **p(C)** | **R** | **EΠ(R;C)** |
| --- | --- | --- | --- |
| **Low=**0 | 0 | 0.3 | 0(0.3)-0=0 |
| **Medium Low=**0.2 | 0.5 | 0.3 | 0.5(0.3)-0.2=0.05 |
| **Medium High=**0.4 | 0.66 | 0.3 | 0.66(0.3)-0.4=-0.2 |
| **High=**0.6 | 0.85 | 0.3 | 0.85(0.3)-0.6=-0.34 |

**Table S2**

**The Firm Expected Profit with Push Incentives: Constant vs Variable Funding**

| **C** | **p(C)** | **R** | **F** | **EΠ_push_(R;C;F)** | **F(C)=C** | **EΠ_push_(R;C;F(C))** |
| --- | --- | --- | --- | --- | --- | --- |
| **Low=**0 | 0 | 0.3 | 1.2 | 0(0.3)-0+1.2=1.2 | 0 | 0(0.3)-0=0 |
| **Medium Low=**0.2 | 0.5 | 0.3 | 1.2 | 0.5(0.3)-0.2+1.2=1.15 | 0.2 | 0.5(0.3)-0.2+0.2=0.15 |
| **Medium High=**0.4 | 0.66 | 0.3 | 1.2 | 0.66(0.3)-0.4+1.2=1 | 0.4 | 0.66(0.3)-0.4+0.4=0.2 |
| **High=**0.6 | 0.85 | 0.3 | 1.2 | 0.85(0.3)-0.6+1.2=0.85 | 0.6 | 0.85(0.3)-0.6+0.6=0.25 |

**Table S3**

**The Firm Expected Profit with Pull Incentives: Constant vs Variable Funding**

| **C** | **p(C)** | **R** | **F** | **EΠ_pull_(R;C;F)** | **F(C)=C** | **EΠ_pull_(R;C;F(C))** |
| --- | --- | --- | --- | --- | --- | --- |
| **Low=**0 | 0 | 0.3 | 1.2 | 0(0.3+1.2)-0=0 | 0 | 0(0.3+0)-0=0 |
| **Medium Low=**0.2 | 0.5 | 0.3 | 1.2 | 0.5(0.3+1.2)-0.2=0.55 | 0.2 | 0.5(0.3+0.2)-0.2=0.05 |
| **Medium High=**0.4 | 0.66 | 0.3 | 1.2 | 0.66(0.3+1.2)-0.4=0.6 | 0.4 | 0.66(0.3+0.4)-0.4=0.07 |
| **High=**0.6 | 0.85 | 0.3 | 1.2 | 0.85(0.3+1.2)-0.6=0.67 | 0.6 | 0.85(0.3+0.6)-0.6=0.17 |

**Table S4**

**The Firm Expected Profit with Mixed Push-Pull Incentives and Constant Funding**

| **C** | **p(C)** | **R** | **F** | **F_push_** | **F_pull_** | **EΠ_mixed_(R;C;F)** |
| --- | --- | --- | --- | --- | --- | --- |
| **Low=**0 | 0 | 0.3 | 1.2 | 0.2 | 1 | 0.2 |
| **Medium Low=**0.2 | 0.5 | 0.3 | 1.2 | 0.2 | 1 | 0.5(0.3+1)=0.65 |
| **Medium High=**0.4 | 0.66 | 0.3 | 1.2 | 0.2 | 1 | 0.66(0.3+1)-0.2=0.66 |
| **High=**0.6 | 0.85 | 0.3 | 1.2 | 0.2 | 1 | 0.85(0.3+1)-0.4=0.70 |

**Table S5**

**The Firm Expected Profit with PAYG Incentives and Constant Funding**

| **C=C_1_+C_2_** | **h(C_1_)g(C_2_)** | **h(C_1_)** | **C_1_** | **C_2_** | **F_push_** | **F_interim_** | **F_pull_** | **EΠ_PAYG_(R;C;F)** |
| --- | --- | --- | --- | --- | --- | --- | --- | --- |
| **Low=**0 | 0 | 0 | 0 | 0 | 0.2 | 0.5 | 0.5 | 0.2 |
| **Medium Low=**0.2 | 0.5 | 0.66 | 0.15 | 0.05 | 0.2 | 0.5 | 0.5 | 0.5(0.8)+0.66(0.45)+0.05=0.75 |
| **Medium High=**0.4 | 0.66 | 0.86 | 0.2 | 0.2 | 0.2 | 0.5 | 0.5 | 0.66(0.8)+(0.86)0.3=0.78 |
| **High=**0.6 | 0.85 | 0.7 | 0.05 | 0.55 | 0.2 | 0.5 | 0.5 | 0.85(0.8)-0.7(0.05)+0.15=0.79 |

**Table S6**

**Relevant R&D Probabilities**

|  | **Target to Hit** | **Hit to Lead** | **Lead Optim.** | **Preclinical** | **Phase I** | **Phase II** | **Phase III** | **Submission to Launch** |
| --- | --- | --- | --- | --- | --- | --- | --- | --- |
| **Transition Probability** | 0,8 | 0,75 | 0,85 | 0,69 | 0,54 | 0,34 | 0,7 | 0,91 |
| **Attrition Rate** | 0,2 | 0,25 | 0,15 | 0,31 | 0,46 | 0,66 | 0,3 | 0,09 |
| **Success Probability** | 0,04 | 0,05 | 0,07 | 0,08 | 0,11 | 0,21 | 0,64 | 0,91 |
| **Variance** | 0,96 | 0,95 | 0,93 | 0,92 | 0,89 | 0,79 | 0,36 | 0,09 |

**Table S7**

**R&D Investments and Compounds Submitted to Launch**

| **% R&D Invest** | 10% | 20% | 30% | 40% | 50% | 60% | 70% | 80% | 90% | 100% |
| --- | --- | --- | --- | --- | --- | --- | --- | --- | --- | --- |
| $\boldsymbol{C}$ | $87,3 | $174,6 | $261,9 | $349,2 | $436,5 | $523,8 | $611,1 | $698,4 | $785,7 | $873 |
| **n°compounds** | 0,11 | 0,22 | 0,33 | 0,44 | 0,55 | 0,66 | 0,77 | 0,88 | 0,99 | 1,1 |
| **p(C)** | 0,23 | 0,41 | 0,55 | 0,65 | 0,73 | 0,79 | 0,84 | 0,88 | 0,90 | 0,92 |
| **p(C)/C** | 0,0026 | 0,0023 | 0,0020 | 0,0019 | 0,0017 | 0,0015 | 0,0014 | 0,0013 | 0,0012 | 0,0011 |

**Table S8**

**Estimates of the Expected Profits without Incentives**

| **% R&D invest** | 10% | 20% | 30% | 40% | 50% | 60% | 70% | 80% | 90% | 100% |
| --- | --- | --- | --- | --- | --- | --- | --- | --- | --- | --- |
| **R=$375** | | | | | | | | | | |
| **Ex. Profit** | $0* | $-20,4 | $-56,3 | $-104,2 | $-161,2 | $-225,3 | $-294,8 | $-368,4 | $-445,3 | $-524,5 |
| **R=$1000** | | | | | | | | | | |
| **Ex. Profit** | $145,4 | $236,6 | $286,3 | $304,2* | $297,5 | $272,1 | $232,3 | $181,4 | $122,1 | $56,2 |
| **R=$4100** | | | | | | | | | | |
| **Ex. Profit** | $867 | $1511,5 | $1958,9 | $2329,6 | $2573 | $2739,5 | $2846,9 | $2909 | $2936,3 | $2937* |

**Table S9**

**Estimates of the Expected Profits with Incentives**

| **% R&D invest** | 10% | 20% | 30% | 40% | 50% | 60% | 70% | 80% | 90% | 100% |
| --- | --- | --- | --- | --- | --- | --- | --- | --- | --- | --- |
| **Push Incentives R=$375 F(C)=$1000** | | | | | | | | | | |
| **Ex. Profit** | $100* | $979,6 | $943,7 | $895,8 | $838,8 | $774,7 | $705,2 | $631,5 | $554,7 | $475,5 |
| **Push Incentives R=$375 F(C)=0,81C** | | | | | | | | | | |
| **Ex. Profit** | $70,7 | $121,04 | $155,8 | $178,7 | $192,3 | $199 | $200,2* | $197,2 | $191,1 | $182,6 |
| **Push Incentives R=$375 F(C)=0,91C** | | | | | | | | | | |
| **Ex. Profit** | $79,4 | $138,5 | $182 | $213,6 | $235 | $251,3 | $261,3 | $267,1 | $269,7 | $269,9* |
| **Pull Incentives R=$375 F(C)=$1000** | | | | | | | | | | |
| **Ex. Profit** | $232,6 | $390,9 | $491,9 | $549,2 | $572,8* | $570,6 | $548,6 | $511,4 | $462,5 | $404,7 |
| **Pull Incentives R=$375 F(C)=0,81C** | | | | | | | | | | |
| **Ex. Profit** | $16,4 | $37,8 | $60 | $80,6 | $98,2 | $112,4 | $122,6 | $129,2 | $132,5 | $132,6* |
| **Pull Incentives R=$375 F(C)=0,91C** | | | | | | | | | | |
| **Ex. Profit** | $18,4 | $44,9 | $74,3 | $103,4 | $130,4 | $154 | $174,2 | $190,7 | $203,8 | $213,7* |
